# Supplementary material for: Chronic Nodular Prurigo: An Update on the Pathogenesis and Treatment
Source: Int J Mol Sci. 2022 Oct 16;23(20):12390. doi: 10.3390/ijms232012390 (PMC9604302; doi:10.3390/ijms232012390)
Supplement: Supplementary file 1 [file ijms-23-12390-s001.zip › ijms-1950426-supplementary.pdf]

Table S1. The inflammatory mediators, which were proposed to participate in the mechanisms of CNPG, and the proportion of AD predisposition in the studies.

**Inflammatory mediators involved in the pathogenesis of CNPG**	**Proportion of patients with history of atopy included in the studies**
**Th2 cytokines**	
Park et al. [26]	Not mentioned
Tokura et al. [27]	No
Fukushi et al. [28]	No
Belzberg et al. [24]	No
**IL-31**	
Hashimoto et al. [36]	Not mentioned
Chaowattanapanit et al. [37]	11.6% patients
**Th17/IL-17, Th22/IL-22**	
Park et al. [26]	Not mentioned
Belzberg et al. [24]	No
Wong et al. [18]	25%
**Endothelin**	
Zhong et al. [19]	45%
Wong et al. [18]	25%
**Nerve growth factor**	
Zhong et al. [19]	45%
